# Clinical outcomes and survivorship of two-stage total hip or knee arthroplasty in septic arthritis: a retrospective analysis with a minimum five-year follow-up

**DOI:** 10.1007/s00264-021-05013-5

**Published:** 2021-03-27

**Authors:** Antonio Russo, Luca Cavagnaro, Francesco Chiarlone, Antonio Clemente, Sergio Romagnoli, Giorgio Burastero

**Affiliations:** 1 Orthopaedic Clinic, Ospedale Policlinico San Martino, Largo R. Benzi 10, 16132 Genova, Italy; 2 Department of Surgical Sciences and Integrated Diagnostic (DISC), University of Genoa, Viale Benedetto XV 6, 16132 Genoa, Italy; 3 Joint Arthroplasty Unit, Orthopaedic and Traumatology 2, Santa Corona Hospital, Viale 25 Aprile 38, 17027 Pietra Ligure, Italy; 4 Department of Orthopedics and Traumatology, CTO Hospital, University of Turin, Via G. Zuretti 29, 10126 Turin, Italy; 5 Prosthetic Surgery Centre, IRCCS Istituto Ortopedico Galeazzi, Via Riccardo Galeazzi 4, 20161 Milan, Italy

**Keywords:** Septic arthritis, Two-stage arthroplasty, Cement spacer, Total hip arthroplasty, Total knee arthroplasty

## Abstract

**Purpose:**

Septic arthritis of the native joint is challenging for orthopedic surgeons because it may lead to wide bone defects and severe impairment of joint function. This study aimed to analyze clinical functional outcomes, the rate of infection eradication, and survival of implants of patients who underwent two-stage arthroplasty for septic arthritis of the hip and knee.

**Methods:**

A retrospective single-centre analysis was conducted of patients treated for septic arthritis of the hip and knee joints through a two-stage surgery between 2012 and 2015. Clinical and radiological records were gathered from the prospectively collected Institutional Arthroplasty Registry. Patients’ pre-operative Harris hip scores and Knee Society scores were compared with those obtained at the latest follow-up. Kaplan–Meier curves were generated to assess survival of implants.

**Results:**

Forty-seven patients were included. The mean follow-up was 85.2 ± 15.4 months. The Harris hip score improved from 39.4 ± 9.9 to 84.5 ± 10.8 points (*p* < 0.001). The Knee Society score improved from 40.7 ± 8.4 to 86.0 ± 7.8 points (*p* < 0.001). Knee Society score-function increased from 25.7 ± 14.2 to 85.4 ± 23.4 points (*p* < 0.001). The infection eradication rates were 92.0% and 90.9% in patients who underwent hip and knee operation, respectively (*p* = 0.891). Overall survivorship of implants after the second stage was 93.6%.

**Conclusions:**

Two-stage arthroplasty provides good to excellent clinical outcomes in cases of active septic arthritis of the hip and the knee, high rates of infection control, and implant survival.

## Introduction

Septic arthritis (SA) is a rare but potentially devastating condition, causing significant pain and disability to affected patients [1, 2]. Its management is challenging, and a multidisciplinary approach is always required. Recently, the incidence of SA has increased because of the aging of the population and the numerous intra-articular procedures performed. Generally, the extent of infection in the setting of SA is limited compared with periprosthetic joint infection (PJI) because of the absence of previously implanted devices. However, since early onset SA can be treated with open arthroscopic debridement, whereas chronic infections need more radical resection and debridement and are associated with wider joint degeneration, the timing of the diagnosis is fundamental [3]. Over the years, several surgical strategies have been proposed to treat evolutive SA. In 1943, Girdlestone described resection arthroplasty, which was demonstrated to efficiently relieve pain and morbidity related to infection; however, leg length discrepancies and poor functional outcomes were common complications of the procedure [4, 5]. More recently, total joint arthroplasty (TJA) has been described as a reliable solution to treat SA with wide joint degeneration to control symptoms and guarantee acceptable restoration of function [6–9]. One-stage TJA has demonstrated good functional outcomes, but the results are still unsatisfactory in terms of eradication in cases of active infection [7, 10, 11].

The use of cement spacers in staged procedures is well established, showing excellent outcomes in joint function and infection eradication [12–15].

The aim of this study was to assess the clinical outcomes of patients treated with two-stage TJA in the setting of SA of the hip and knee at the medium-term follow-up. Endpoints of this analysis were clinical and functional outcomes, expressed as clinical scores of validated questionnaires, rate of eradication of the infection, and survivorship of the implants in this cohort of patients.

## Materials and methods

The local institutional review board approved this single-center study. Informed consent was obtained from all individual participants included in the study.

### Data collection and inclusion criteria

A retrospective analysis of our prospectively collected Institutional Arthroplasty Registry was conducted from January 1st, 2012 to January 1st, 2015, searching for patients treated for SA. Patients suffering from SA of the hip or the knee joint treated with two-stage TJR with at least five years of follow-up and who gave their written informed consent were considered eligible for the study. Patients with primary SA, SA following previous surgery, and post-infiltrative SA were all included in the analysis. Patients who suffered from PJI, had incomplete clinical data, or were missing at follow-up were excluded. Smoking status, age at time of surgery, and comorbidities were not considered exclusion criteria for the study.

The diagnosis of SA was made based on one or a combination of the following parameters: clinical signs of infection (oedema, erythema, functional limitation, a draining sinus communicating with the joint), elevated serum C-reactive protein ([CRP] > 5 mg/dL) and erythrocyte sedimentation rate ([ESR] > 30 mm/h) values, radiographic findings of bone resorption and loss of articular space, finding of intra-operative purulence, and positive intra-operative or synovial fluid microbiology. General characteristics of the study populations, such as age, sex, body mass index (BMI), American Society of Anesthesiologists (ASA) score, follow-up, interval between the two stages, and pathogens involved were retrieved and are presented in Tables 1 and 2.

**Table 1:** Demographic characteristics of patients included in the study

		Hips *n* = 25 (%)	Knees *n* = 22 (%)
Sex	M	13 (52)	12 (54.5%)
	F	12 (48)	10 (45.5%)
Mean age (±SD), y		56.4 (±15.0)	55.3 (±13.9)
Mean BMI (±SD), kg/m^2^		25.7 (±4.6)	27.2 (±4.1)
ASA	I	10 (40)	11 (50)
	II	7 (28)	6 (27.3)
	III	8 (32)	5 (22.7)
Side	L	19 (76)	16 (72.7)
	R	6 (24)	6 (27.3)
Aetiology	Post-surgery	4 (16)	13 (59.1)
	Post-infiltrative	2 (8)	4 (18.2)
	Primary	19 (76)	6 (27.3)
Main comorbidities	DM	7 (28)	5 (22.7)
	Drug abuse	6 (24)	4 (18.2)
	HIV	5 (25)	3 (13.6)
	HCV	4 (16)	4 (18.2)
	Systemic TBC	1 (4)	–
	CVD	8 (32)	6 (27.3)
	CPD	4 (16)	5 (22.7)
	Epilepsy	2 (8)	1 (4.5)
Mean interstage interval (±SD), w		14.5 ± 2.9	14.9 ± 2.8
Mean follow-up (±SD), m		86.7 ± 16.0	85.6 ± 15.1

*ASA*, American Society of Anesthesiology; *CPD*, chronic pulmonary disease; *CVD*, cardiovascular disease; *DM*, diabetes mellitus; *F*, female; *HCV*, hepatitis C virus; *HIV*, human immunodeficiency virus; *L*, left; *M*, male; *m*, months; *n*, numbers; *R*, right; *SD*, standard deviation; *TBC*, tuberculosis; *y*, years

**Table 2:** Pathogens identified in preoperative microbiological analysis of synovial fluid

	Hips, *n* (%)	Knees, *n* (%)
MSSA	7 (28)	6 (27.3)
MRSA	3 (12)	1 (4.5)
CoN staphylococci	–	3 (13.6)
*Streptococcus* sp.	1 (4)	2 (9.1)
*Pseudomonas* sp.	2 (8)	1 (4.5)
*Mycobacterium* sp.	2 (8)	2 (9.1)
*E. coli*	1 (4)	–
*Proteus* sp.	1 (4)	–
Polymicrobial	2 (8)	2 (4.5)
Culture negative	6 (24)	5 (22.7)

*MRSA*, methicillin-resistant *Staphylococcus aureus*; *MSSA*, methicillin-sensible *Staphylococcus aureus*; *CoN*, coagulase-negative; *sp.*, species

### Clinical and radiographic assessment

The clinical and radiographic assessments of patients were conducted pre-operatively and after the second stage at one, three and six months. Then, patients were evaluated once per year. Clinical evaluation of patients treated for SA of the hip included Harris hip score (HHS) [16] evaluation and physical examination. Radiographic assessment included an anteroposterior (AP) view of the pelvis and AP and lateral views of the hip. Similarly, patients who suffered SA of the knee underwent physical examination, Knee Society score (KSS) [17] evaluation, KSS for function (KSS-F), and anteroposterior and lateral radiographs of the knee (Table 3). Radiographic images were assessed by two orthopedic fellows (A.R., A.C.) preoperatively and at final follow-up; these assessments aimed to identify loosening, radiolucent lines, malposition, component migration, leg length discrepancy (LLD), hip stem subsidence, and heterotopic ossifications. The Brooker classification was used to classify hip heterotopic ossification [18]. Radiological evaluation of the knees was carried out according to the Knee Society total knee arthroplasty radiographic evaluation for long-stemmed revision prostheses to fully evaluate the entire lengths of the prostheses [19]. Disagreements were resolved via discussion among all the authors.

**Table 3:** Clinical functional outcomes

	Hips (*n* = 25)	*p*-value
Mean HHS (±SD)		
Pre-operative	39.4 ± 9.9	<0.001
Final	84.5 ± 10.8	
Mean offset (±SD)		
Pre-operative	51.1 ± 5.0	0.31
Final	52.0 ± 4.6	
Post-operative LLD	7.4 ± 7.0	
	Knees (*n* = 22)	*p*-value
Mean KSS (±SD)		
Pre-operative	40.7 ± 8.4	<0.001
Final	86.0 ± 7.8	
Mean KSS-F (±SD)		
Pre-operative	25.7 ± 14.2	<0.001
Final	85.4 ± 23.4	

*HHS*, Harris hip score; *KSS*, Knee Society score; *KSS-F*, Knee Society score-function; *LLD*, leg length discrepancy; *SD*, standard deviation

*p*-value is relative to the comparison of preoperative values and those obtained at latest follow-up

### Surgical procedures

All the two-stage procedures were performed by a single surgeon experienced in complex septic TJA surgery (G.B.). All the hips were approached through a posterolateral incision, whereas all the knees were operated on using a medial parapatellar approach. During the first-stage surgery, a femoral head and acetabulum or knee resection was performed, along with extensive debridement of the surrounding tissues involved in the infectious process. Three to six samples of septic tissue were withdrawn for cultures. Subsequently, joints were irrigated with 10 L of an antiseptic solution, and a preformed articulating antibiotic-loaded cement spacer containing gentamicin and vancomycin was positioned. The degree of bone defect was defined by the senior author at the time of surgery using the Paprosky [20, 21] and the Anderson Orthopedic Research Institute (AORI) [22] classifications (Table 4). At least two weeks of intravenous antibiotic therapy was administered. Then, the switch to oral antibiotic therapy or targeted intravenous antibiotic therapy for a period of at least four weeks was decided on based on the microbiological results.

**Table 4:** Classification of bone defects

		Hips *n* = 25 (%)
Paprosky acetabulum	I	7 (28.0)
	IIA	6 (24.0)
	IIB	5 (20.0)
	IIIA	6 (24.0)
	IIIB	1 (4.0)
Paprosky femur	I	20 (80.0)
	II	5 (20.0)
		Knees *n* = 22 (%)
AORI femur	I	9 (40.9)
	IIA	4 (18.2)
	IIB	5 (22.7)
	III	4 (18.2)
AORI tibia	I	8 (36.4)
	IIA	5 (22.7)
	IIB	4 (18.2)
	III	5 (22.7)

*AORI*, Anderson Orthopedic Research Institute

During the second stage, the spacer was removed, and a further debridement was performed. Three to six samples were retrieved for microbiological analysis, as was a specimen for frozen section histology. In case of persistence of infection, a spacer exchange was performed. Depending on the case, the senior surgeon decided how to manage each bone defect and which type of prosthetic design to implant.

### Post-operative pathway

A specific antibiotic therapy was prescribed until the results of the intra-operative microbiology were received and continued thereafter when needed. On the second post-operative day the surgical drain was removed, and patients were encouraged to engage in partial weight-bearing with a walker or crutches. Thromboembolism prophylaxis was started on the same day as the surgery with heparins and compressive stockings, and it was continued for at least 45 days. Celecoxib 200 mg twice daily was administered to patient who had no contraindications for 20 days post-operatively for the prevention of heterotopic ossifications.

### Statistical analysis

Statistical analysis was conducted using IBM SPSS Statistics version 26.0 (IBM Corp., Armonk, NY, USA). Categorical variables were expressed as the number of events or percentages. Continuous variables were expressed as the mean ± standard deviation (SD). Pre-operative clinical score values and at final follow-up were compared using the paired *t*-test. Kaplan–Meier survival curves were provided to analyze the success rate of treatments. Treatment success was defined as infection eradication, absence of major complications during the interstage period that required spacer exchange or conversion to TJA, or any additional surgery. Statistical significance was set at *p* < 0.05 for all the analyzed variables.

## Results

Forty-seven consecutive patients (25 male, 22 female) met the inclusion criteria and were enrolled in the study. Of these, 25 (53.2%) suffered from SA of the hip and 22 (46.8%) had SA of the knee. The overall mean age at stage one was 55.9 ± 14.3 years. The overall mean BMI was 26.4 ± 4.4 kg/m^2^. All the patients had been reimplanted at a mean of 14.7 (range, 10.7 to 22.9) weeks after the first stage. The mean follow-up was 85.2 ± 15.4 months. The most common comorbidities were chronic cardiovascular diseases (29.8%) and diabetes mellitus (25.5%). A history of intravenous drug abuse (21.3%), HIV (17.7%), and HCV (17.0%) were other frequently encountered conditions in this series. Seventeen (36.2%) patients had previous surgery to the affected joint and 6 (12.8%) had previous infiltrative therapies; in 24 (51.1%) cases, the infection was classified as primary SA. Details on demographics, ASA scores, comorbidities, and general characteristics of the study population are presented in Table 1.

### Pathogens

Methicillin-sensible *Staphylococcus aureus* (MSSA) was the most common pathogen found at microbiology (13 cases, 27.7%). Methicillin-resistant *Staphylococcus aureus* (MRSA) was identified in four cases (8.5%), as were coagulase-negative staphylococci (CoN-St) and *Mycobacterium tuberculosis*. Polymicrobial and culture-negative infections were encountered in 4 (8.5%) and 11 (23.4%) cases, respectively. The complete list of pathogens identified preoperatively is shown in Table 2.

### Clinical and functional outcomes

The mean HHS significantly improved from 39.4 ± 9.9 points pre-operatively to 84.5 ± 10.8 points at final follow-up (*p* < 0.001). Differences between offset on the healthy contralateral side (51.1 ± 5.0 mm) and on the operated side at final follow-up (52.0 ± 4.6 mm) were not considered statistically significant (*p* = 0.31). The mean LLD was 7.4 ± 7.0 mm.

The mean KSS improved from 40.7 ± 8.4 points pre-operatively to 86.0 ± 7.8 points at final follow-up (*p* < 0.001). The mean KSS-F improved from 25.7 ± 14.2 points to 85.4 ± 23.4 points (*p* < 0.001).

### Complications

The overall complication rate was 27.7%. Major complications that required revision or reoperation were reported in 14.9% of cases.

In two cases (4.3%), patients underwent spacer exchange for persistency of infection. Of these, one patient was diagnosed with primary *Mycobacterium tuberculosis* SA of the hip and the other had a polymicrobial infection of the knee.

Two patients (4.3%) underwent hip spacer dislocation, which was managed through revision TJA with good results. One patient (2.1%) had positive intra-operative microbiology at the time of the second stage and underwent suppressive antibiotic therapy with good results.

One case of aseptic loosening that required revision (2.1%) of the knee after final implantation was registered. One patient (2.1%) had infection recurrence in the hip after the second stage, which required prosthesis removal and a Girdlestone procedure.

Five (10.6%) patients developed heterotopic ossification of the hip, visible on radiographs. However, the ossifications were non-progressive, the patients remained asymptomatic, and no further treatment was needed.

One patient (2.1%) with prior internal fixation of the femur with a plate suffered from extensive damage to the knee extensor mechanism, which required reconstruction through the medial gastrocnemius flap during the second stage. One patient (2.1%) who underwent hip operation developed haematoma after spacer implantation, which required surgical drainage.

One patient (2.1%) who underwent hip surgery suffered from a previous multifocal diaphyseal fracture of the femur with subsequent neurapraxia of the sciatic nerve and had poor HHS at final follow-up (48 points). No complications related to the SA and its treatment were responsible for the poor score.

### Eradication rate and survival analysis

**Fig. 1:**
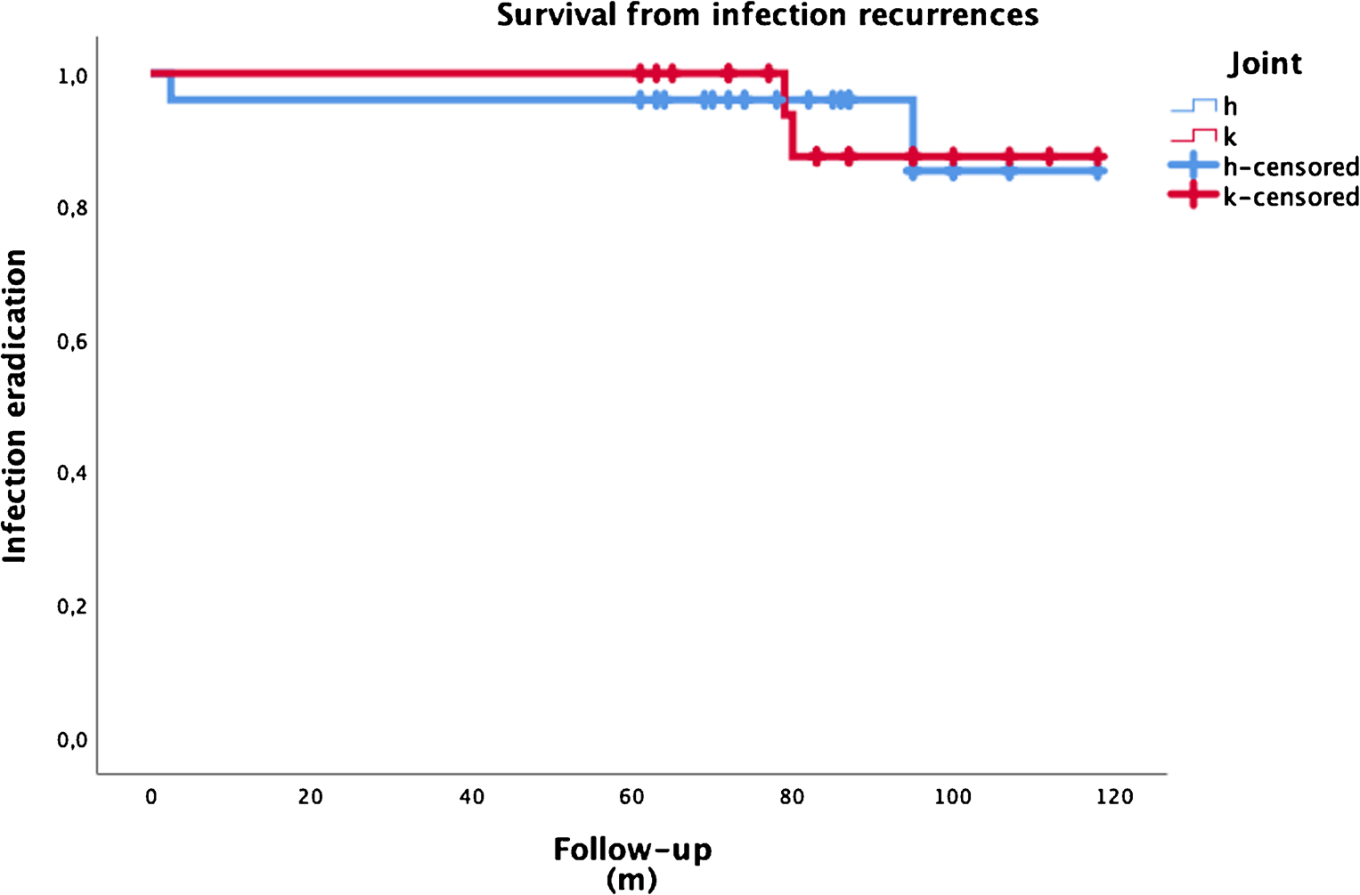
Survival curve of two-stage procedures free from septic recurrences. H, hips; K, knees; m, months

Eradication of the infection was defined as the absence of any infection recurrence after spacer implantation or second-stage surgery. The overall eradication rate was 91.5%. Eradication rate was 92.0% in patients who underwent hip surgery and 90.9% in patients who underwent knee operation. This difference was not statistically significant (*p* = 0.891). Figure 1 displays the survival curve for septic complications.

**Fig. 2:**
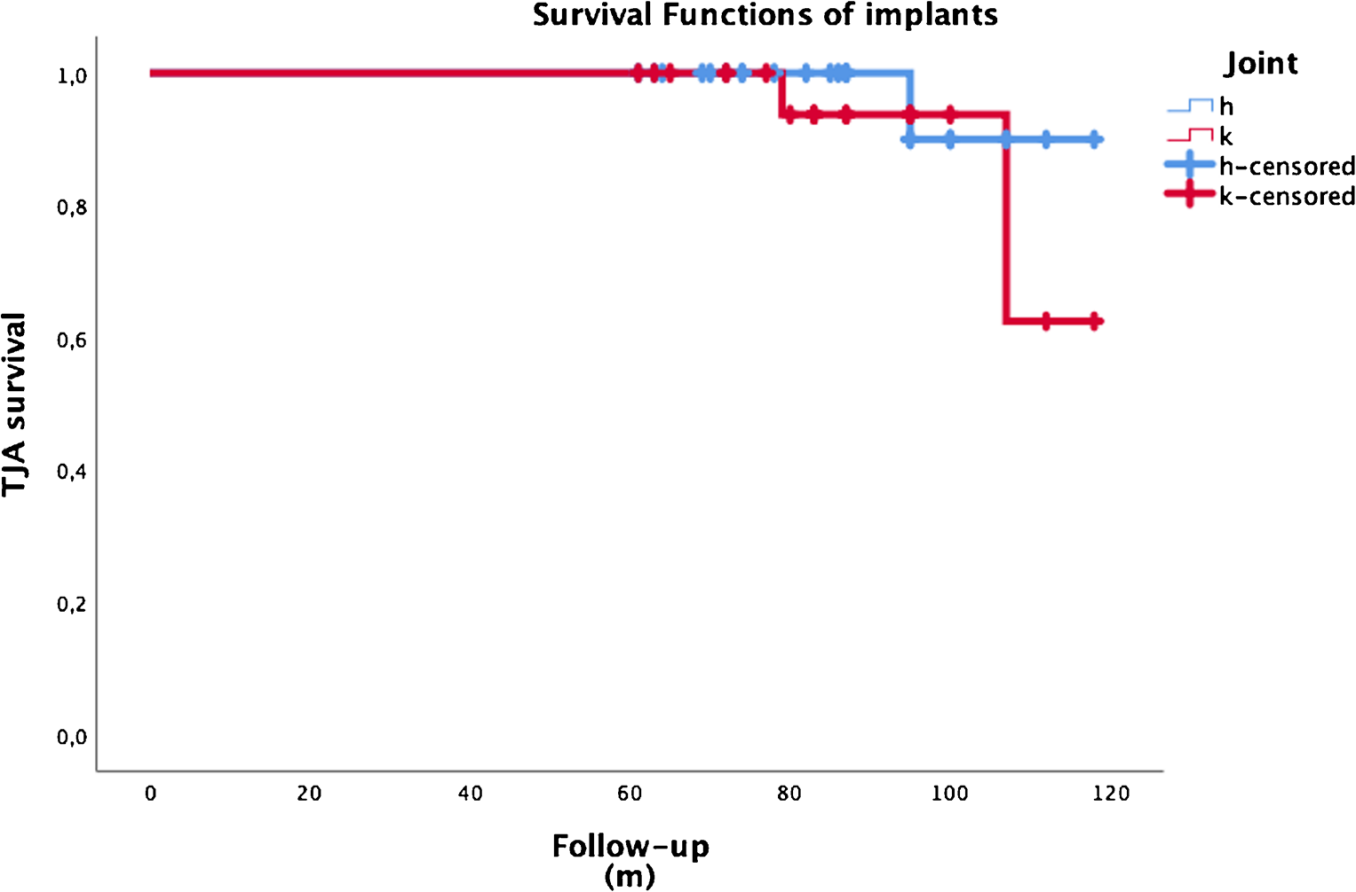
Survival curve of implants free from septic and mechanical complications. H, hips; K, knees; m, months; TJA, total joint arthroplasty

Overall survivorship of implants after the second stage was 93.6%. The survivorships of hip and knee implants were 96.0% and 90.9%, respectively. The difference observed in terms of implant survival rate between hip and knee prosthesis was not considered statistically significant (*p* = 0.447). The survival curves of prosthetic implants after the second stage are presented in Fig. 2.

## Discussion

The appropriate surgical approach to evolutive SA associated with extensive joint degeneration continues to be debated. One-stage replacement was proposed as a viable technique to manage patients with SA of the hip and knee, but the risk of increased PJI is still concerning [7, 11]. Moreover, one-stage procedures are not indicated in cases of active infection. In these cases, two-stage replacement may be considered the treatment of choice to recover function and avoid infection recurrences [14, 23]. Papanna et al. [10] applied a differentiated protocol based on active versus quiescent SA, which was treated using two-stage versus one-stage replacement, respectively. In this cohort of 18 patients, no recurrence of infection or implant failures were registered at a mean 70-month follow-up. Anagnostakos et al. [24] treated 16 patients with two-stage replacement, achieving 88% control of infection. In general, the available literature demonstrated that two-stage procedures after SA provided good to excellent clinical results, with success rates ranging from 84 to 100% [6, 12, 15, 25, 26]. The results of this study demonstrated overall primary infection control in 92% of hips and 90.9% of knees and an overall implant survival of 93.6% after the second stage. Notably, patients developing SA are often affected by specific comorbidities (diabetes mellitus, intravenous drug abuse) that are well-known risk factors for PJI and generalized sepsis. From this point of view, a staged approach is safer and more advisable for chronic SA management.

Scores obtained at final follow-up from HHS, KSS, and KSS-F were classified as good to excellent. Two poor results were registered in patients with prior post-surgical sequelae, which affected the final scores, as mentioned in the “Results” section. These results are comparable with those available in the literature.

Different types of spacers have been used in the setting of two-stage surgery; some authors have used prefabricated spacers, while others have used handmade cement spacers or cement beads [10, 26–28]. However, cement spacers seem to guarantee better functional outcomes during the period between stages when compared with cement beads [9, 14, 29, 30]. In 2019, Li et al. [31] conducted a comparative analysis on patients who received two-stage exchange arthroplasty following SA; patients in group I underwent the Girdlestone procedure and subsequent TJR, whereas patients in group II were implanted with a cement spacer at the first stage. The presence of the spacer effectively maintained leg length and simplified the second surgery, decreasing blood loss and operative time. Based on the specific bone defect encountered in the first stage, we used a preformed antibiotic-loaded cement spacer loaded with vancomycin and gentamicin. A handmade acetabular spacer was always coupled to the preformed spacer in the SA of the hips [13]. In knees, reinforced stems were incorporated into the preformed spacer when needed to guarantee more stability. In our cohort of patients, such parameters as LLD and offset did not demonstrate any significant difference from pre- to post-operative values, corroborating the assumption that spacers can efficiently preserve the length and appropriate muscular tension of the treated limb. However, two cases (4.3%) of hip spacer dislocation were registered.

Although two-stage arthroplasty is a reliable procedure, surgeons should consider related complications, such as mortality, which is higher compared to that associated with other treatments (e.g., one-stage surgery), and mechanical or septic complications that the patient could undergo during the interstage period [32–34]. In a recent study, Xu et al. [30] found that older age, infection by a resistant pathogen, and high pre-operative levels of CRP were independent predictors of treatment failure after two-stage exchange arthroplasty for SA. At the same time, they found that serum ESR, CRP, and interleukin 6 (IL-6) had no benefits in predicting infection persistency before second-stage prosthesis implantation. In our series, in four cases (8.6%), patients had complications related to the spacer, which were successfully managed with spacer exchange in two (4.3%) cases and conversion to TJA in the other two (4.3%) cases, with optimal final outcomes.

**Fig. 3:**
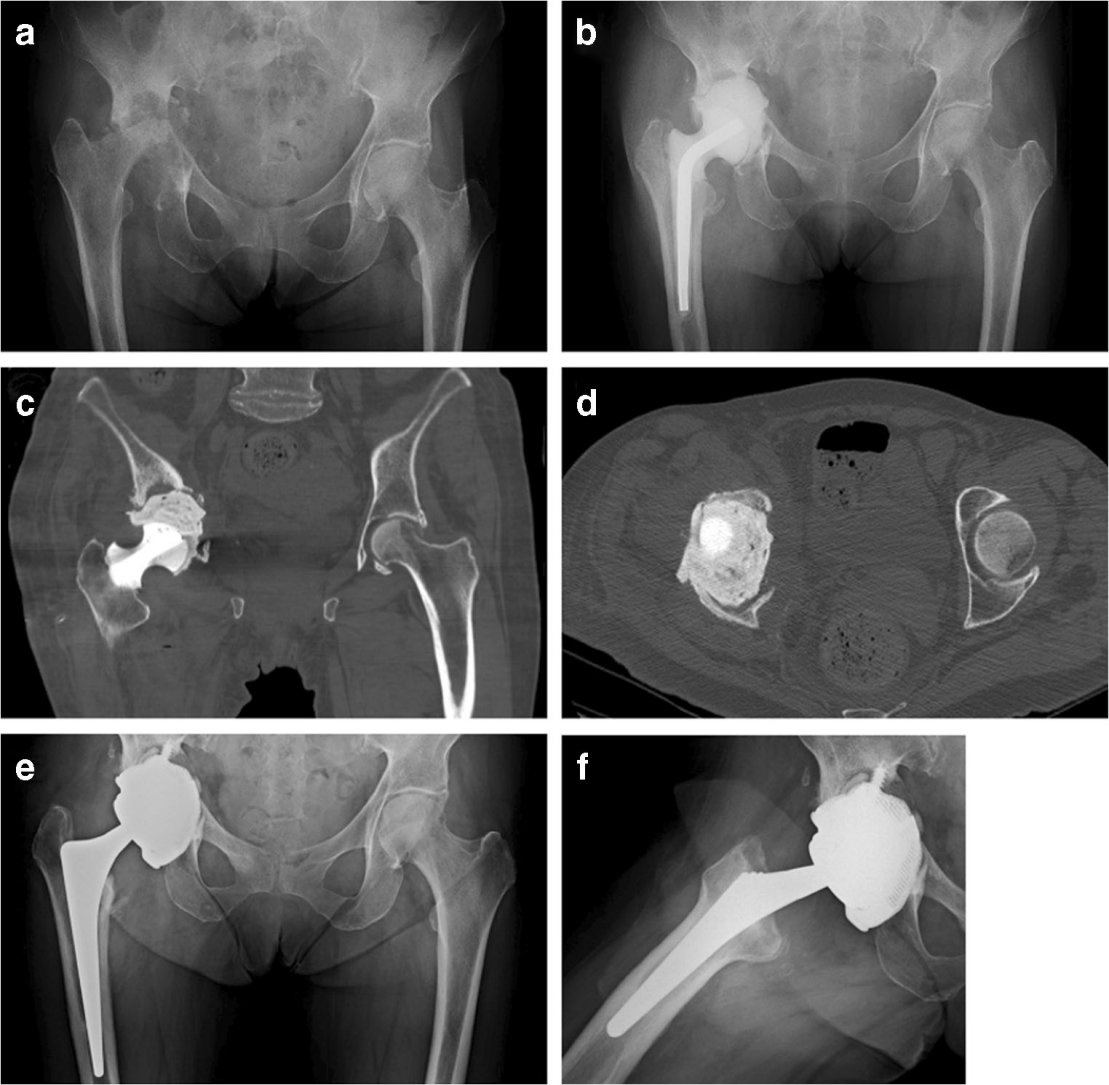
Images of a 76-year-old female patient suffering from polymicrobial SA of the right hip. **a** AP view of the pelvis showing Paprosky type IIIB acetabular bone defect and Paprosky type I bone defect on the femur. **b** AP radiograph of the pelvis 2 months after articulating cement spacer implantation. On the femur, a prefabricated cement spacer was used, whereas a hand-molded acetabular spacer was used on the acetabular side. **c**, **d** AP and axial CT details of acetabular bone defect. **e**, **f** AP and lateral views of the right hip at final follow-up. A single wedge cementless stem was used, and the extensive acetabular defect was managed through a custom-made component

**Fig. 4:**
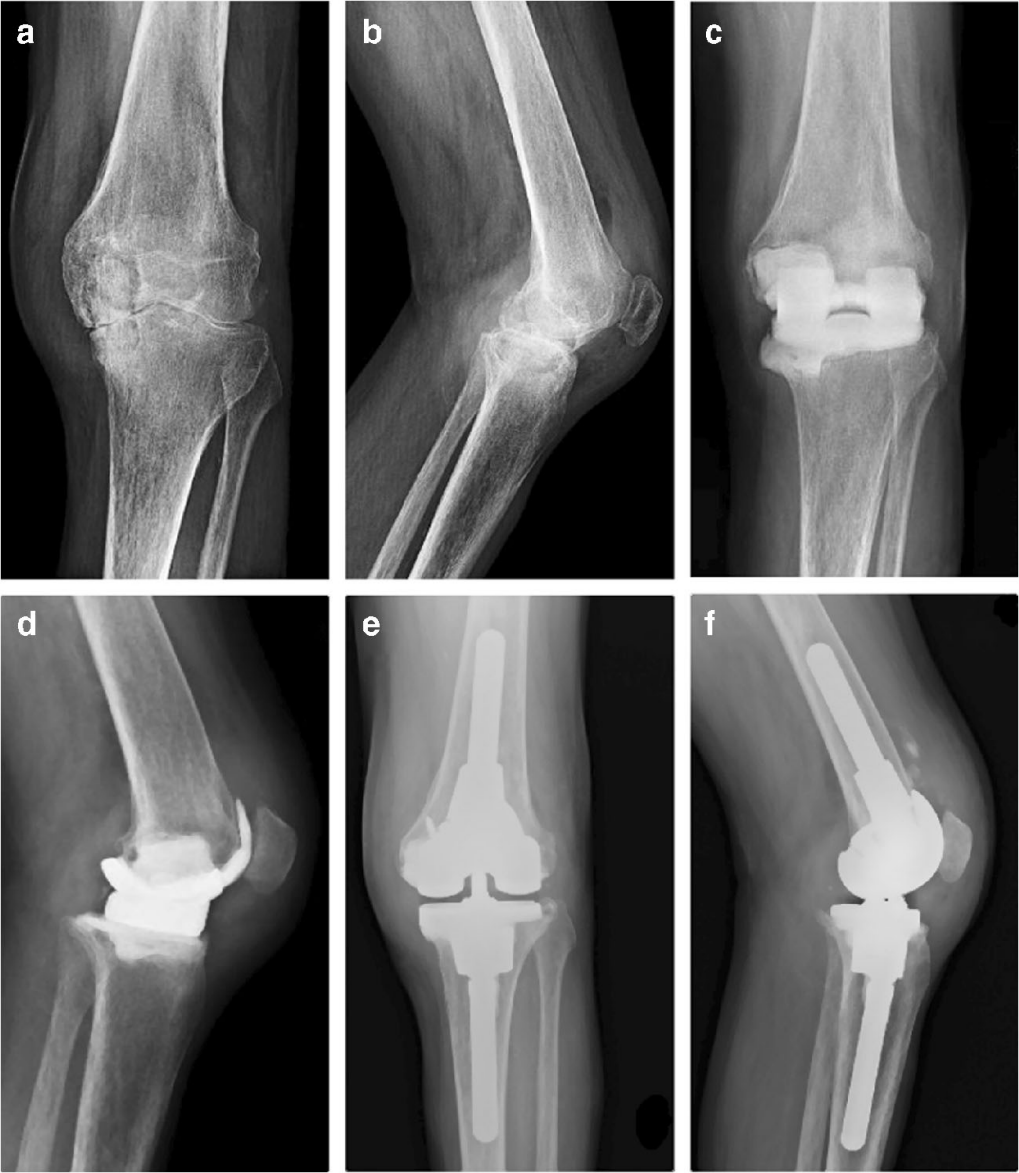
Images of a 64-year-old male patient suffering from a tubercular SA of the left knee. **a**, **b** AP and lateral view of the left knee demonstrating the wide degenerative process and deformity resulting from the infection. **c**, **d** AP and lateral view of the knee 3 months after the prefabricated articulating cement spacer was positioned showing a wide cavitary bone defect on the medial femoral condyle. **e**, **f** AP and lateral radiographs at final follow-up. The medial femoral condyle was reconstructed using a tantalum acetabular augment

Chronic SA often massively impairs bone stock, especially on the acetabular side (Fig. 3). The subsequent necessary debridement leads to massive bone loss in hips and in knees. The surgeon should be aware of this feature of SA and should always be prepared to manage complex bone defects (Fig. 4). In such surgical situations, an extremely accurate spacer implantation tailored to a patient’s bone defects should prevent further bone loss and interstage complications.

It is important to highlight that several limitations affect this study. The retrospective nature of the analysis contains inherent limitations that must be considered when evaluating results. Although we applied our institutional two-stage surgery protocol to all the patients included, the type of spacer and antibiotic therapy were individualized, and this could have resulted in a bias in the analysis. The absence of control groups made any considerations on different treatment options impossible, and the small sample size limits the statistical power of this analysis. Different types of SA (post-infiltrative, post-surgery, primary) were all analyzed together, representing a diagnostic bias of our analysis. Finally, no further investigations (e.g., PET) have been performed during follow-up to exclude late-onset chronic infections and confirm a disease-free condition. However, the strengths of this study were the prospective collection of data and relatively long follow-up (>5 years), as well as that all the patients underwent a standardized protocol of treatment and follow-up and diagnoses and surgery were performed in a standard manner by the same surgeon.

## Conclusions

Two-stage arthroplasty provides good to excellent clinical outcomes in cases of active SA of the hip and the knee, as well as high percentages of infection control and implant survival. During the interstage period, cement spacers maintained adequate biomechanical parameters of the affected limbs. More high-quality case-control studies are needed to clarify which is the best treatment in the setting of SA.
